# Somniloquism

**Published:** 1887-03

**Authors:** 


// HALL'S
### JOURNAL OF HEALTH

TRUTH DEMANDS NO SACRIFICE; ERROR CAN MAKE NONE.

VOL. 34. MARCH, 1887. No. 3.

### SPECIAL.

We have continued to send the JOURNAL to a number of our subscribers whose prepaid terms expired with the close of the last year, since which the greater number have renewed their subscriptions Some few, however, have not done so, and unless renewals are made in such cases before the issue of our next number their names will be omitted from our mailing list hereafter.

### SOMNILOQUISM.

In the February number of the JOURNAL, we introduced the subject of somnambulism, giving some individual instances, illustrative of the continued activity of the intellectual faculties, working by rule and method in unison with the physical organism, at periods of profound sleep.

Somniloquism is a phenomenon which signifies, as its Latin derivation—*somnus* and *loquor*—would imply, *talking* in one's sleep. It is the most frequent of occurrence of all manifestations attendant upon a state of somnolence, being no less common to children than to adults, and holds so near relationship with somnipathy, or the mesmeric sleep, that the one has been properly designated *nataral* and the other *sympathetic* or induced somnolence. The former is often brought about by over-exertion or a disturbed mental condition, whilst the latter is common to a class of minds easily brought under the influence of the mesmerist, known as sensatives. That the condition, in either case, is substantially the same has been shown by a variety of experiments. Perty, in his "Mystical Revelations," gives an account of a natural somniloquist, the wife of a well-known gentleman, who, in her sleep, conversed with ease and fluency whenever her husband took hold of her hand. In this state, she made exact revelations of the thoughts and designs of those present, and not unfrequently foreshadowed the events that were to transpire on the day following. This goes to show that one in a state of somniloquy is sometimes endowed with super-mundane gifts, and is able to perceive what is passing in other minds, and to compass not only the present but the future.

It is, indeed, conceded by able writers upon the subject, that persons in the comatose state evince remarkable powers of perception and a range of ideas altogether beyond the intellectual plane of the subject, and what is more, wholly out of range of the operator, as in the instance of the lady in question.

The author of the well known lyric drama, "La Somnambula," makes the beautiful sleep-walker lisp the name of her lover, and talk of a lost ring, whilst wandering from her couch, all unconsciously, in the still hours of night. So, too, the great master of dramatic art pictures Lady Macbeth as audibly betraying the secret of a great crime, whilst walking in her sleep.

> "Unnatural deeds
> Do breed unnatural troubles; Infected minds
> To their deaf pillows will discharge their secrets."

Those who are greatly engrossed in some intellectual pursuit are quite apt to give vent to it in the hours of repose. The late Prof. Brittan in "Man and his Relations," remarks upon this as follows: "It is especially when some subject engages all the faculties, or events of great moment weigh heavily on the mind at the close of the day, that we are most likely to give utterance to what is passing in the mind, during the hours of sleep," and he mentions the case of a clergyman who was accustomed to extemporize his discourses, and, whilst asleep, frequently conudcted the entire religious services, according to the formula of his church, including singing, preaching from a text and praying. On one occasion he engaged the attention of his fellow passengers on a steamboat in this manner, greatly to their edification if not to their profit.

Professor Brittan was himself addicted to somniloquism in a remarkable degree, during that period of his life devoted to lecturing in public, and sometimes awoke to find himself surrounded by interested listeners to lengthy discourses delivered with a force and eloquence which surpassed his normal efforts, in the same direction, at least we have been so informed by those who were privileged to take note of them. In remarking upon these occurences, Doctor Brittan says: "It was after adopting the clerical profession—which made it necessary to address public assemblies—that this practice of extemporizing in sleep assumed an orderly form, and began to awaken a lively interest in the minds of such persons as chanced to be my auditors." In youth he had been addicted to somnambulism, but an accidental fall down a flight of stairs served to put an end to these nocturnal wanderings.

Muler, in his "Physiology of the Senses, Voice, Muscular Motion," etc., says: "The connection between ideas and movements is sometimes as close as that between different ideas; thus, when an idea and a movement have frequently occurred in connection with each other, the idea often excites the involuntary production of the movement."

It is by this connection between the idea and the muscular action, rather than the exercise of will, that Muler and a number of other writers upon psychological subjects, whose opinions have been received as authority, account for physical manifestations attendant upon the dreamy state, including sleep-walking, talking and singing.

A curious case is reported of an officer engaged in the expedition to Louisburg, in 1758, which shows how completely the sleeper is subject to the will of one who is in rapport with him. One day, finding him asleep on a locker, they made him believe that he had fallen overboard, and that a shark was pursuing him; and upon being entreated to dive for his life, the officer threw himself with great force on the cabin floor. At another time, after the landing of the army at Louisburg, his friends found him asleep one day in his tent, evidently much disturbed by the cannonading. They then made him believe he was engaged in actual combat, whereupon he expressed great fear and a disposition to run away. They remonstrated, but increased his fears by imitating groans, and when he asked who had been hit, they named his particular friends, and told him at length that his nearest comrade had fallen, when he sprang to the ground, rushed out of the tent, and ended his dream by falling over the tent ropes. On another occasion, they conducted him through a quarrel, which ended in a duel; and a pistol being placed in his hands, he fired at the word, and was awakened by the report. Of these and similar transactions he preserved no recollection.

Prof. Joseph Rodes Buchanan, an account of whose remarkable discoveries in relation to the brain was given in the November number of the Journal, performed many remarkable experiments with sensitives, in presence of members of the medical faculty and other distinguished persons, for the purpose of illustrating the complete subjection of the subject to the will of the operator, and the ease with which certain faculties of the mind could be enlivened by the mere touch of its phrenological locality or key, if we may use the expression.

It is well known that the magnetic sleepcr, whether natural or induced, will, in most instances, converse freely with his sympathetic companion or magnetizer. Lorry mentions the case of a girl, ten years old, that would make long speeches in her sleep when her mother placed one hand on her head, but who became silent when the hand was removed. Professor Agardh, of Sweden, met with a boy who, in the sympathetic sleep, could speak Latin with great fluency and also converse in French and English, although ignorant of those languages, conversing with him when they were introduced. Some interesting experiments were made with Jenny Lind, the Swedish nightingale, and a mesmeric sensitive, some years ago, which were reported in the Manchester *Courier*, and extensively republished by the American press; the sensitive keeping pace with, rather than repeating after her, some Swedish and Italian songs in the most perfect manner in relation to both words and music, yet having had but little instruction in music, and none whatever in the language employed.

Fernel also gives an account of an uneducated boy who was able to speak Greek and Latin when in the magnetic sleep, and such instances are more common than the reader might suppose.

Dr. George Moore, author of "The Power or the Soul over the Body," (English) says: "We see, we feel, we hear, according to a power apart from sense, or not necessarily associated with it, and according to a nature that may see, hear, feel, in a different manner with different instruments, or even immediately, that is, without instruments, and rather according to the state of the will than the state of the body."

Strangely enough this author has permitted himself to make use of a very careless expression in speaking of "sleep-walking and sleep-talking," which describes them as "forms of insanity." It would, indeed be as strange as it would be cruel to adjudge one insane for displaying traits *abnormal*, simply because *unusual*, in periods of sleep.

It is a truth, if we mistake not, that young children never become victims of insanity, yet they are oftentimes addicted to talking in their sleep, especially after undergoing unusual fatigue or excitement, which acts as a stimulant to the ever active mind. And, after all, what is it otherwise than voicing our dreams; shaping our midnight fancies into words that, oftentimes, have no meaning for the common ear? To make these the criterion of mental poise would be like passing sentence upon the vagaries of a dream, no more to be estimated by rigid rules of plain common sense than are the fantastic creations of the poet's brain, whose rhythmic numbers march with the heart's tenderest emotions, into realms far removed from the common place.

"As angels in some brighter dreams,  
Call to the soul when man doth sleep,  
So some strange thoughts transcend our wonted themes,  
And into glory peep."

